# Resistance of freshwater sediment bacterial communities to salinity disturbance and the implication for industrial salt discharge and climate change-based salinization

**DOI:** 10.3389/frmbi.2023.1232571

**Published:** 2023-11-13

**Authors:** Helen Tammert, Carmen Kivistik, Veljo Kisand, Kairi Käiro, Daniel P. R. Herlemann

**Affiliations:** ^1^ Centre for Limnology, Chair of Hydrobiology and Fisheries, Estonian University of Life Sciences, Tartu, Estonia; ^2^ Institute of Technology, University of Tartu, Tartu, Estonia; ^3^ Department of Biological Oceanography, Leibniz Institute for Baltic Sea Research Warnemünde, Rostock, Germany

**Keywords:** Baltic Sea, sea level rise, littoral, salinization, pulse disturbance, experiment, industrial discharge management

## Abstract

The impact of salinization on freshwater ecosystems became apparent during the 2022 ecological disaster in the Oder River, located in Poland and Germany, which was caused by salt discharge from mining activities. How bacterial communities respond to salinization caused by industrial salt discharge, or climate change-driven events, depends on the sensitivity of these complex bacterial communities. To investigate the sensitivity of bacterial communities to pulse salinization, we performed an experiment in the salinity range from 0.2 to 6.0. In addition, we sampled similar salinities in the littoral zone of the Baltic Sea where the bacterial communities are permanently exposed to the aforementioned salinities. To simulate a major disturbance, we included an ampicillin/streptomycin treatment in the experiment. Although the addition of antibiotics and increase in salinity had a significant impact on the water bacterial richness and community composition, only antibiotics affected the sediment bacterial community in the experiment. In contrast, sediment bacterial communities from the Baltic Sea littoral zone clustered according to salinity. Hence, sediment bacterial communities are more resistant to pulse changes in salinity than water bacteria but are able to adapt to a permanent change without loss in species richness. Our results indicate that moderate pulse salinization events such as industrial salt discharge or heavy storms will cause changes in the water bacterial communities with unknown consequences for ecosystem functioning. Sediment bacterial communities, however, will probably be unaffected in their ecosystem functions depending on the disturbance strength. Long-term disturbances, such as sea level rise or constant salt discharge, will cause permanent changes in the sediment bacterial community composition.

## Introduction

Climate change is responsible for weather extremes that can cause salinity pulse shocks to freshwater environments during storm events or long-term salinization due to sea level rise (IPCC, 2022). Moreover, changes in evaporation and precipitation result in the salinization of freshwater in inland waters (Jeppesen et al., 2020). Another source of salinization is the often neglected industrial pollution caused by mining activities, which are the main sources of river salinization in Europe (Bäthe and Coring, 2011). The discharge of hypersaline effluents into freshwater ecosystems can increase the salinity levels in a matter of a few hours or days to brackish conditions (Johnson et al., 2010), which in turn affects the ecosystem structure and functioning (Sauer et al., 2016). Hypersaline discharge was also the key factor for the 2022 ecological disaster of the Oder River, located in Poland and Germany, which caused the death of hundreds of tons of fish along a 500-km stretch of the river due to the sudden growth of toxic brackish water algae (Free et al., 2023). The increase in salinity causes extreme conditions for freshwater bacterial communities, as microorganisms face particular barriers when transitioning to another salinity level (Logares et al., 2009). The salt concentration of the environment has been reported to be one of the main factors shaping the distribution of aquatic bacterial communities (Crump et al., 2004; Herlemann et al., 2011; Fortunato et al., 2012). Despite the sensitivity of water bacterial community composition to changes in salinity, a constant species richness has been found in different salinities for water bacteria (Herlemann et al., 2011) and sediment bacteria in the Baltic Sea (Klier et al., 2018). This is in contrast to observations of eukaryotic richness where lower numbers of organisms in intermediate salinities (i.e., 6–10) for eukaryotes were found (Remane, 1934; Olli et al., 2023).

One of the fundamental goals of microbial ecology is to understand to what extent environmental disturbance is accompanied by changes in bacterial richness and community diversity. Disturbances that lead to changes in the community composition (beta diversity) and richness (alpha diversity) may alter the functioning of the community and affect the ecosystem processes (Berga et al., 2017). The recovery of the community after compositional reduction or change depends on the strength and the duration of the disturbance, as well as the dispersal of the taxa and recruitment from the existing seedbank. In conclusion, the reaction of the community members to the disturbances is a combination of past and present events (Hillebrand and Blenckner, 2002; Andersson et al., 2014; Renes et al., 2020; Philippot et al., 2021). In the littoral environment, microbial communities both in the water and in the sediment upper layer are exposed to unstable and inhomogeneous sets of biological and physical conditions that cause a complex mixture of growth-influencing factors. The diverse range of ecological niches has created seedbanks for dynamic and flexible communities, with the potential to inhabit a high number of rare species (Shade et al., 2012). The response of community members to disturbance depends on the frequency and intensity of the disturbance (Berga et al., 2012). Small-scale and possibly recurring changes in the environment, such as inter-seasonal chemical–biological fluctuations, are expected to cause minor modulations in the bacterial diversity and the presence of niche specialists favored by certain abiotic or biotic factors (Andersson et al., 2014). Strong pulse disturbances (e.g., industrial salt discharge or extreme weather events) and continuous long-term pressures (e.g., sea level rise, evaporation, precipitation, and industrial salt leakage) can lead to more severe disturbances in the bacterial community. In general, bacterial communities have shown to be sensitive to disturbance and are usually unable to recover to their original state (Renes et al., 2020). The flexibility of bacterial physiology and the energetic cost of adaptation mechanisms to withstanding disturbance can determine the resilience and resistance level of bacterial community members. For aquatic environments, the vast majority of the disturbance studies have shown sensitivity to temperature, salinity, and acidification disturbance of bacterial communities (Andersson et al., 2014; Renes et al., 2020; Seidel et al., 2023).

In this study, we investigate the responses of littoral freshwater sediment and water bacterial communities to a pulse increase in salinity in manipulation experiments. The results are compared with the bacterial community composition in natural brackish sites with similar salinities. Our hypotheses were that an increase in salinity (i) significantly alters the bacterial community composition and brackish-tolerant bacteria dominate in the water and sediment, and (ii) does not affect the bacterial richness of freshwater sediment and water bacterial communities.

## Materials and methods

The top 0- to 2-cm layer of sandy sediment, at a water depth of 0.5 m, and corresponding water were collected for the freshwater salinization experiment from the littoral zone of Lake Võrtsjärv, Estonia. The sediment was sieved through 0.5-mm mesh filter to remove larger debris, homogenized, and then distributed to 10-L aquaria in a 3- to 4-cm-thick layer. To the sediment, freshly 85-µm mesh-filtered Lake Võrtsjärv water was added. The aquaria also contained snails (*Ampullaceana balthica*), pebbles, and small stones with biofilm. The snails and their microbiome were analyzed in the study of Kivistik et al. (2022). In total, 13 × 10 L aquaria were divided into four different treatments: three reference aquaria without further manipulation (REF); three aquaria with an addition of 5 mg/L of ampicillin and 5 mg/L of streptomycin (AB); three aquaria where the salinity of the water was increased to 3 (SAL3, oligohaline); and four aquaria where the salinity of the water was increased to 6 (SAL6, mesohaline). For salinization, we used commercially available Reef Salt (AquaMedic) containing 1,000 mg/L sodium, 1,200 mg/L magnesium, 420 mg/L calcium, 350 mg/L potassium, 19,700 mg/L chloride, 2,200 mg/L sulfate, 180 mg/L carbonate, and 16 mg/L strontium. The experimental aquaria were constantly supplied by air and held at 16.1°C –17.5°C for 8 days. The water and sediment samples were taken on days 1, 3, 6, and 8 of the experiment. For water samples, 100 mL of water from each aquarium, for a total of 55 samples, was filtered through 0.2-µm membrane filters (Whatman) and frozen at –80°C. In addition, 3 g of sediment was sampled from each aquarium, for a total of 53 samples, shock frozen in liquid nitrogen, and stored at –80°C. The temperature, oxygen, pH, and salinity were measured every day with a YSI ProDSS multiparameter meter.

The natural site water and sediment samples were collected in the Baltic Sea coastal area in Estonia on 17 and 18 June 2019. The triplicate water and surface sediment samples were collected at the natural sites from a 0.5- to 1-m depth (*n* = 42) at similar salinities as in the experiment. The *in situ* freshwater sampling sites were Selja Pond (FP), and the freshwater (FW) rivers Selja River (SR) and Kunda River (KR). The sampling site with a salinity of 3 (oligohaline; SAL3) was Selja Bay (SB). The sampling sites with a salinity of 6 (mesohaline; SAL6) were Nõva (NÕ), Ristna West (RW), and Ristna East (RE) (**Table 1**).

**Table 1:** Environmental variables in natural sampling sites.

Sampling site	Salinity	Salinity class	Temperature (°C)	DO (mg/L)	pH	Latitude (°) Longitude (°)
Kunda River (KR)	0.3	Freshwater (FW)	17.6	10.23	8.55	59.546306 N 26.650722 E
Selja River (SR)	0.3	Freshwater (FW)	19.9	10.85	8.72	59.555118 N 26.343352 E
Selja Pond (FP)	0.3	Freshwater (FW)	23.8	5.8	8.11	59.509762 N 26.538798 E
Selja Bay, Baltic Sea (SB)	2.5	Oligohaline (SAL3)	19	12.45	8.93	59.554670 N 26.340361 E
Nõva site, Baltic Sea (NÕ)	6.3	Mesohaline (SAL6)	16.5	7.46	7.71	59.554553 N 26.339870 E
Ristna East, Baltic Sea (RE)	6.5	Mesohaline (SAL6)	20.7	14.05	8.87	59.270542 N 23.738648 E
Ristna West, Baltic Sea (RW)	6.5	Mesohaline (SAL6)	19.9	7.62	8.03	59.271705 N 23.734715 E

### DNA extraction and sequence processing

The DNA from the experimental water samples and the DNA and RNA of the sediment and natural site water samples were extracted according to the modified protocols from Lueders et al. (2004) and Weinbauer et al. (2002). For the water filters dichlorodimethylsilane-treated glass beads (three beads with a diameter of 3 mm, and 0.5 g of beads with diameter 0.5 mm) were added to 2-mL tubes. Cell lysis was performed in 750 µL of 120 mM NaPO_4_ buffer (pH 8) and 250 µL of TNS solution [500 mM Tris-HCl pH 8.0, 100 mM NaCl, and 10% sodium dodecyl sulfate (SDS) (weight to volume (wt/vol)], and the samples were bead beaten for 3 min at 2,000 rpm (revolutions per minute) using a Mikro-dismembrator U (B. Braun Biotech International, Melsungen, Germany). After a 1-h incubation at 65°C, we applied a second bead beating for 3 min at 2,000 rpm, which was followed by centrifugation at 14,000 rpm for 5 min. The supernatant was transferred to a new 2-mL tube and a mixture of phenol : chloroform : isoamyl alcohol (25 : 24 : 1) at pH 8 was then added and carefully mixed. The phases were separated by centrifugation at 14,000 rpm for 5 min and the upper aqueous phase was placed in a new 2-mL tube. For purification of both DNA and RNA samples, 1 volume of chloroform : isoamyl (24 : 1) was added and mixed carefully. After centrifugation at 14,000 rpm for 12 min, nucleic acids in the upper aqueous phase of sediment samples were divided equally between two new 1.5-mL tubes: one for DNA and another for RNA. For the removal of the RNA from the DNA sample, 2 µL of RNase (100 mg/mL; QIAGEN, Venlo, the Netherlands) was added and incubated at 37°C for 30 min. The nucleic acids were precipitated by incubating at room temperature for 15 min with a 0.7 volume of cold isopropanol. The samples were then centrifuged at 14,000 rpm for 20 min. The resulting pellet was washed with 250 µL of 95% ethanol, centrifuged at 14,000 rpm for 5 min, and then dried at 50°C after ethanol removal (≈ 5–15 min). The pellet was resuspended in a 50 µL of elution buffer (10 mM Tris-HCl, 0.5 mM EDTA, pH 9.0; QIAGEN).

To remove the DNA from the RNA samples, DNase treatment was performed by using the TURBO DNA-free™ Kit (Invitrogen, Thermo Fisher Scientific, Waltham MA, USA), in accordance with the manufacturer’s protocol. Both the amount and quality of the nucleic acids were measured with a NanoDrop™ UV–Vis spectrophotometer (Thermo Fisher Scientific, Waltham MA, USA). The iScript™ Select cDNA synthesis kit (Bio-Rad, Hercules, CA, USA) was used to transcribe the RNA of the sediment samples into cDNA, in accordance with the manufacturer’s protocol.

For the bacterial community analysis, a two-step PCR protocol was used. In the first step, DNA and cDNA were PCR amplified using the primers Bakt_341F and Bakt_805R (Herlemann et al., 2011), as described by Kivistik et al. (2020). In the second step, sample-specific tags were added to the first step’s PCR products. The amplicons were purified with PCR Kleen™ (Bio-Rad, Hercules, CA, USA). The sequencing was conducted, at FIMM at the University of Helsinki, Finland, with Illumina sequencing using PE250 chemistry.

The sequences were quality checked using Trimmomatic (V0.36) (Bolger et al., 2014) to remove any Illumina-specific sequences and regions with low-sequence quality (i.e., with an average quality score < Q20). The PCR primer sequences were removed using the default values in Cutadapt (V2.3) (Martin, 2011). The reads were paired (16-bp overlap, with a minimum length of 300 bp) and quality trimmed using the VSEARCH tool (Rognes et al., 2016). The sequences were then taxonomically assigned using the SILVA next-generation sequencing (NGS) pipeline (Glöckner et al., 2017), which was based on the SILVA release version 138.1 (released 2020). SILVA NGS was used to perform additional quality checks according to SINA-based alignments (Pruesse et al., 2012) with a curated seed database in which the PCR artifacts or non-small subunit (SSU) reads are excluded. The longest read served as a reference for taxonomic classification using a BLAST (version 2.2.30+) search against the SILVA SSURef dataset. The classification of the reference sequence of each cluster (98% sequence identity) was then mapped to all members of the respective cluster and to their replicates. Samples with less than 1,000 reads were removed. Non/bacterial sequences, such as chloroplastic, mitochondrial, eukaryotic, and archaean, were excluded because the primer set employed in the analysis had only a very limited coverage of these groups. This resulted in 7,422,343 sequences for 197 samples. The raw reads were deposited at the National Center for Biotechnology Information (NCBI)’s Sequence Read Archive (SRA) under bioproject number PRJNA724976, accession number SAMN18865857-SAMN18866052.

### Statistical analysis

For alpha diversity measures, the data were rarefied with bootstrapping using Explicet (Robertson et al., 2013) and expressed as Chao1 and Shannon index values. Sampling events with at least three parallel samples were tested by analysis of variance (ANOVA) (natural sites) and repeated measures ANOVA (experiment) combined with *post-hoc* Tukey’s pairwise test to calculate the significant differences between the taxonomic richness using the PAST software package (version 4.12; Hammer et al., 2001). For beta diversity, the data were cumulative sum scaling (CSS) normalized by the R package metagenomeSeq (Paulson et al., 2013). The differences in the bacterial community composition were tested by multivariate permutational analysis of variance (PERMANOVA) in natural samples and by repeated measures PERMANOVA in the experiment. ANCOVA was used to compare the slopes in the ordination axis–salinity plots using PAST. Community composition was visualized by principal coordinate analysis (PCoA). PERMANOVA and PCoA were both based on the Bray–Curtis dissimilarity, as implemented in the PAST software package version 4.12 (Hammer et al., 2001) using read abundances normalized with CSS. The Molbiol Tools’ online Multiple List Comparator (https://molbiotools.com/) was used to analyze the similarity of the bacterial communities (Jaccard index) and for visualization of similarity as Venn diagrams.

## Results

### Bacterial richness

**Figure 1:**
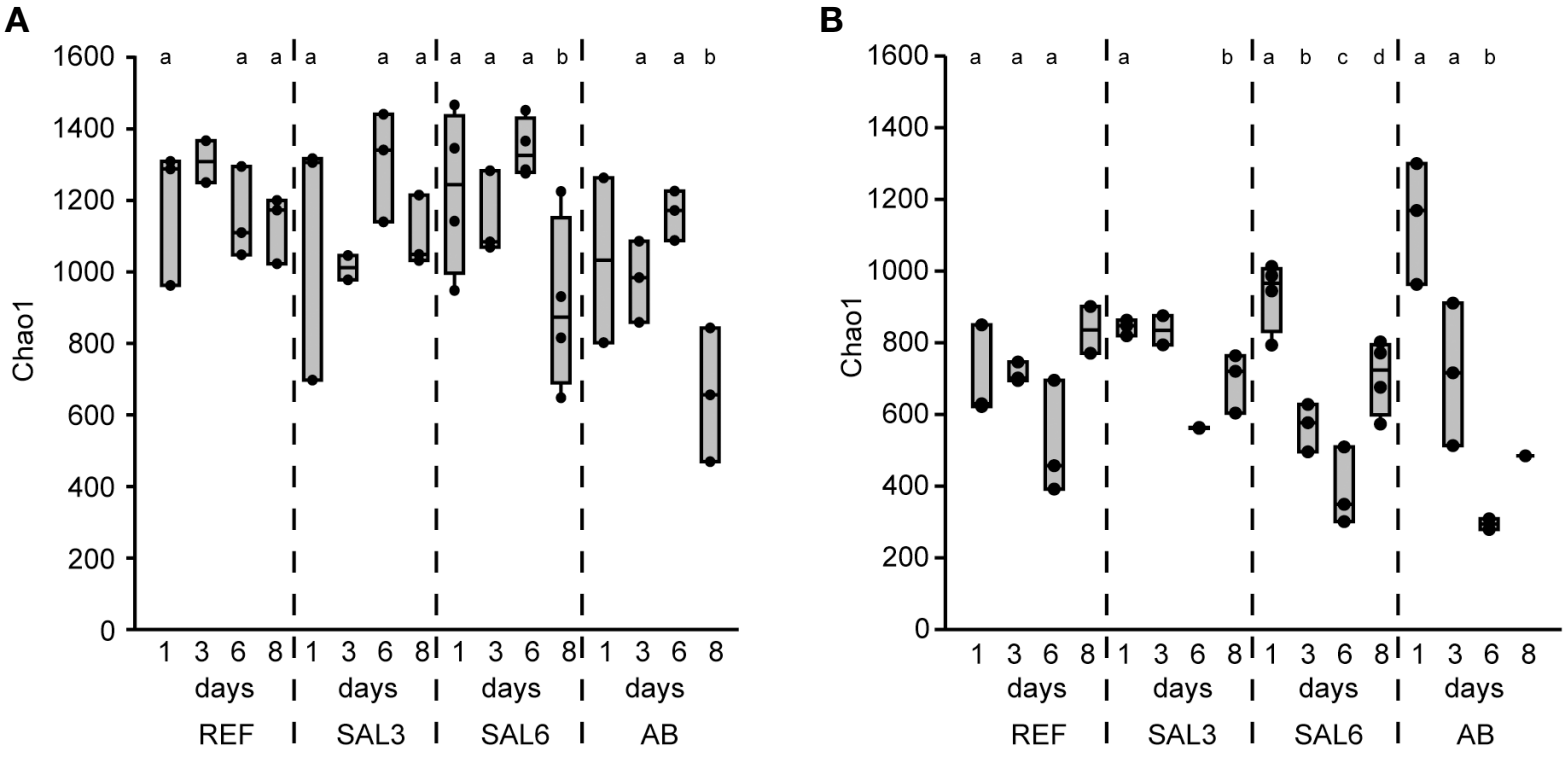
The bacterial richness based on DNA represented by the Chao1 richness estimates of the reference and treatments during the time course of the experiment. **(A)** Sediment; and **(B)** water. REF, reference aquaria; SAL3, salinity increased to 3; SAL6, salinity increased to 6; AB, antibiotic treatment. The non-capital letters (a and b) above the box plots indicate statistical significance within one group, which are separated by the dashed lines.

**Figure 2:**
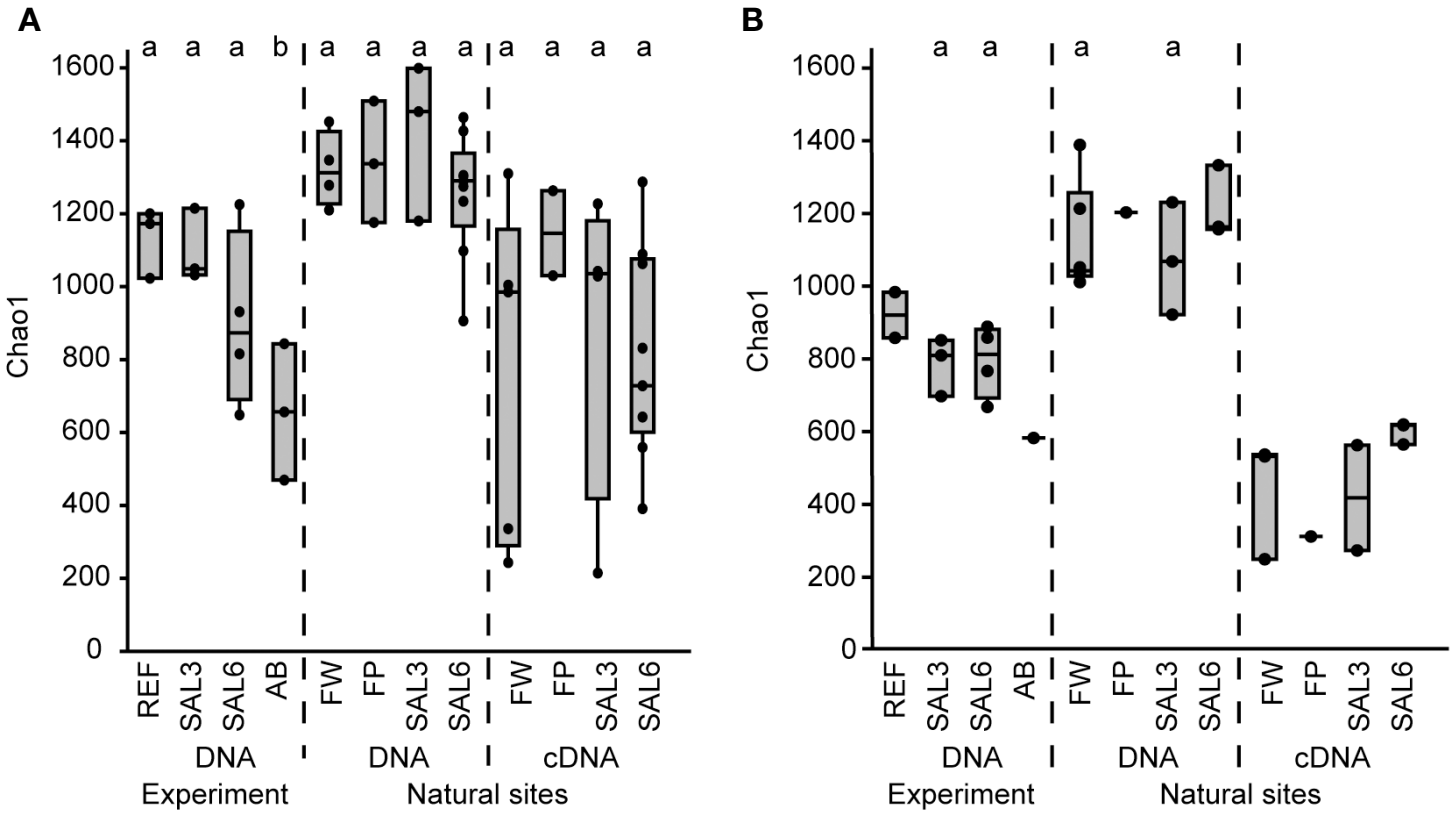
The bacterial Chao1 richness estimates based on the DNA and cDNA on the last day (day 8) of the experiment and in the natural sites. **(A)** Sediment; and **(B)** water. Experiment: REF, reference aquaria; SAL3, salinity increased to 3; SAL6, salinity increased to 6; AB, antibiotic treatment. Natural sites: FW, freshwater; FP, freshwater pond; SAL3, salinity 3; SAL6, salinity 6. The non-capital letters (a, b, and c) above the box plots indicate statistical significance within one group, which are separated by the dashed lines.

The sediment bacterial Chao1 richness estimate showed insignificant changes in the reference aquaria (REF: Chao1 1184, SE ± 40.0) and salinity 3 (SAL3: Chao1 1142, SE ± 63.7) treatment during the 8 days of the experiment (**Figure 1A**). The salinity 6 (SAL6: Chao1 1156, SE ± 61.6) and antibiotic treatment (AB: Chao1 979, SE ± 76.4) reduced the sediment bacterial richness in the DNA-based analysis (**Figures 1A**, **2A**) after day 6 of the experiment (Tukey’s test, *p* < 0.05). One of the salinity treatments was significantly different from the reference (**Figure 2A**). A similar pattern was observed for the cDNA-based analysis, but too few samples were available for statistical analysis. In contrast, the bacterial richness in the water samples showed an intense dynamic by decreasing for day 6 but recovering by the end of the experiment (**Figure 1B**). In the salinity 6 treatment, water bacterial Chao1 richness estimates decreased significantly (Tukey’s test, *p* < 0.05) during the experiment from 935 (SE ± 49.1) to 387 (SE ± 63.0) and recovered at day 8 to a lower level (Chao1 705, SE ± 51.7), compared with the beginning of the experiment (Tukey’s test, *p* < 0.05). In the antibiotic treatment, a similar pattern to the water bacterial community was observed: the Chao1 richness estimate decreased significantly from 1,144 (SE ± 98.4) on day 1 to 294 (SE ± 15.0) on day 6 (Tukey’s test, *p* < 0.05), and recovered for day 8. The Shannon index values showed a similar pattern to the Chao1 richness estimates for water and sediment bacterial communities (**Supplementary Figures 1A, B**).

The differences in the sediment bacterial richness in natural site rivers and the coast of the Baltic Sea (i.e., FW, FP, SAL3, and SAL6 sites) and also between the experiment (i.e., REF, SAL3, and SAL6) and the natural sites were insignificant for the DNA-based analysis (**Figure 2A**). The Chao1 richness estimate and Shannon index revealed similar patterns, whereas the cDNA of the experiment did not contain enough samples for statistical analysis (**Supplementary Figures 2A, 3A**). The comparison of experiment day 8 and the natural sediment bacterial communities with the same salinity revealed that half of the operational taxonomic units (OTUs) were shared, whereas 35% were specific to natural SAL3 and 42% to SAL6 sites (**Supplementary Figure 4**). In the experiment fewer specific OTUs were observed than in the natural sites (16% in SAL3 and 12% in SAL6). A significantly higher Chao1 richness estimate was observed for only sediment DNA than with the cDNA in the natural sites for SAL6 (Tukey’s test, *p* < 0.05; **Figure 2A**).

The water bacterial community in the natural sites had little variation in the taxonomic richness, the Chao1 estimate was 1,045 (SE ± 63.6) in freshwater rivers, 994 (SE ± 92.7) in the salinity 3 site, 1,144 (SE ± 59.9) in the salinity 6 sites, and 1,129 in the freshwater pond (**Figure 2B**). The cDNA-based Chao1 richness estimate was lower in all sites: 336 (SE ± 98.8) in FW, 314 (SE ± 150.5) in SAL3, 503 (SE ± 18.7) in the SAL6 sites, and 203 in the FP. The Shannon index of the water samples was comparable between DNA and cDNA (**Supplementary Figure 3B**).

In the water, the majority of OTUs were specific to natural sites, and 42% were shared between experiment day 8 and the natural sites for SAL3 and SAL6 (**Supplementary Figure 5**). Similar to the low number of specific OTUs observed in the sediment of the experiment, a low number of specific OTUs were also found in the water of the experiment (12% SAL3 and 9% SAL6) compared with the natural sites.

### Bacterial community composition

The sediment and water bacterial community composition at the phylum/class level was dominated by Gammaproteobacteria (20.4%–33.6%), Alphaproteobacteria (9.3%–24.5%), and Bacteroidetes (8.8%–22.7%) in both the experiment and natural sites (**Supplementary Figure 6**).

**Figure 3:**
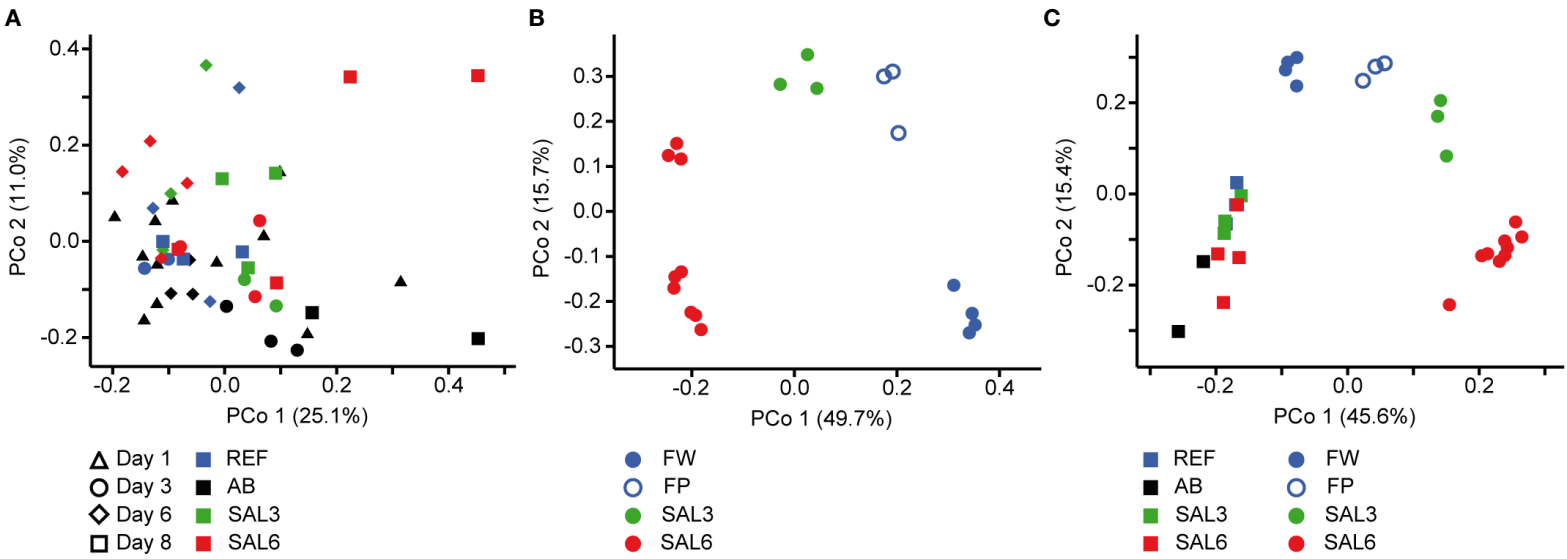
Principal coordinate analysis of the bacterial community composition in the sediment based on the DNA **(A)** during the experiment, **(B)** in the natural sites, and **(C)** on the last day of the experiment (day 8) and in the natural sites with the same salinity. Experiment: REF, reference aquaria; SAL3, salinity increased to 3; SAL6; salinity increased to 6; AB, antibiotic treatment. Natural sites: FW, freshwater; FP, freshwater pond; SAL3, salinity 3; SAL6, salinity 6.

**Figure 4:**
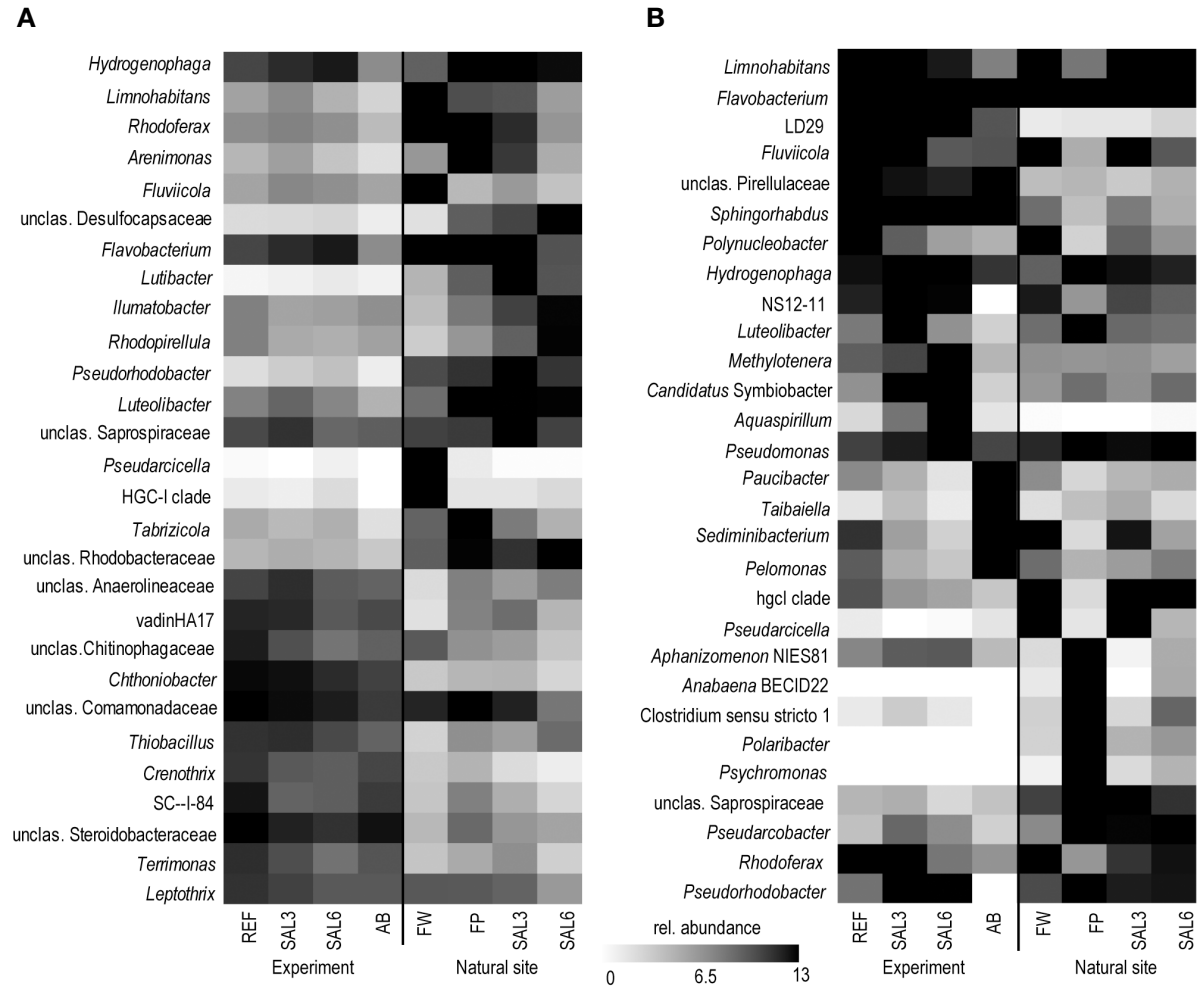
Most abundant OTUs on the last day of the experiment and in the natural sites based on DNA. **(A)** Sediment; and **(B)** water. Experiment: REF, reference aquaria; SAL3, salinity increased to 3; SAL6, salinity increased to 6; AB, antibiotic treatment. Natural sites: FW, freshwater; FP, freshwater pond; SAL3, salinity 3; SAL6, salinity 6. OTU, operational taxonomic unit.

On the finest phylogenetic level (OTU), the sediment bacterial community composition in the treatments showed high variability on PCoA (**Figure 3A**). The AB treatment differed significantly from the salinity treatments and reference (repeated measures PERMANOVA, *p* = 0.007, *R* ^2^ = 49.0%). The SAL6 treatment did not show significant changes in the bacterial community composition during the first 6 days of the experiment. At the end of the experiment (i.e., day 8), the bacterial communities were separated into two clusters on the PCoA plot. Both clusters were represented by two replicates out of the four, with differences from the reference for one set (repeated measures PERMANOVA test, *p* = 0.009, *R* ^2^ = 92.5%). The SAL3 treatment was not different from the REF. The cDNA-based analysis showed no difference in the active fraction of the bacterial community in any treatments (PERMANOVA, *p* > 0.05). On the basis of sediment DNA, sequences from *Hydrogenophaga*, *Flavobacterium*, unclassified Saprospiraceae, unclassified Anaerolineaceae, vadinHA17, unclassified *Chthoniobacter*, *Chthoniobacter*, unclassified Comamonadaceae, *Thiobacillus*, *Crenotrix*, SC-I-84, unclassified Steroidobacteriaceae, *Terriomonas*, and *Leptothrix* were among the most abundant bacteria in all the samples on any day of the experiment (**Figure 4A**). In the antibiotic treatment, the abundance of several OTUs decreased.

**Figure 5:**
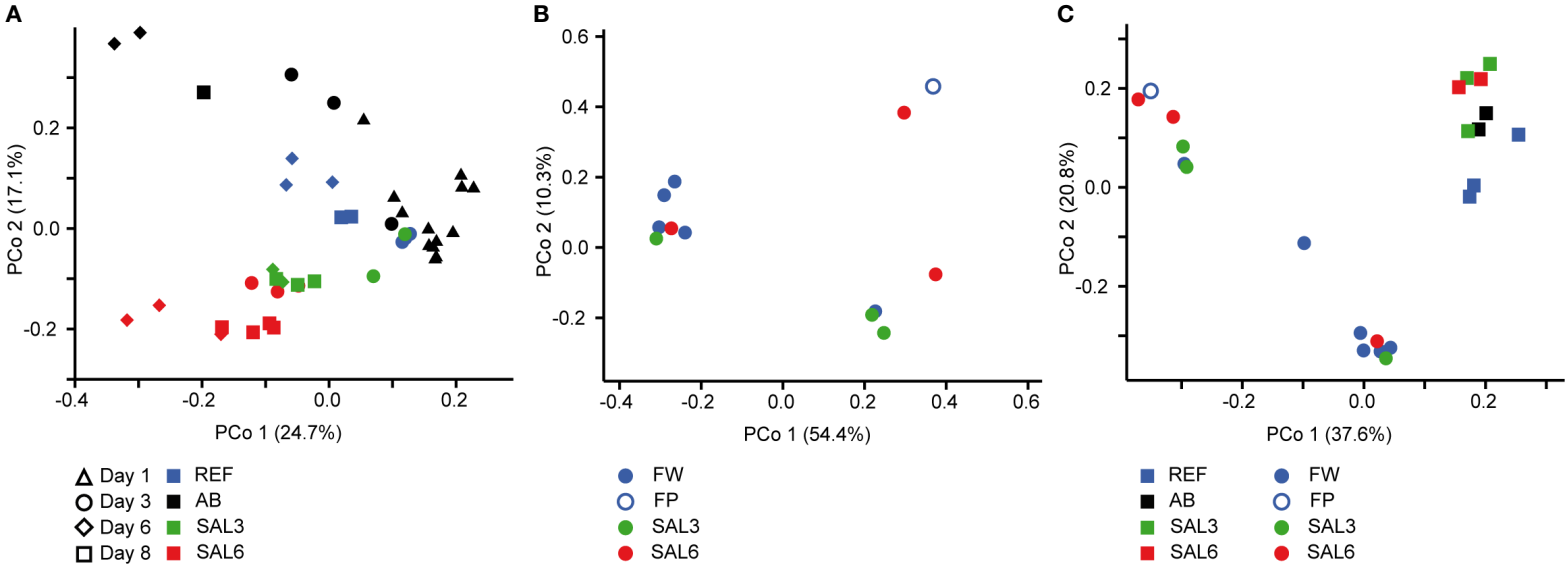
Principal coordinate analysis of the bacterial community composition in the water based on the DNA **(A)** during the experiment, **(B)** in the natural sites, and **(C)** on the last day of the experiment (day 8) and in the natural sites with the same salinity. Experiment: REF, reference aquaria; SAL3, salinity increased to 3; SAL6, salinity increased to 6; AB, antibiotic treatment. Natural sites: FW, freshwater; FP, freshwater pond; SAL3, salinity 3; SAL6, salinity 6.

Artificial salinization and the addition of antibiotics caused a significant shift in the water bacterial community composition on the finest phylogenetic level (repeated measures PERMANOVA test, *p* = 0.0001, *R* ^2^ = 73.7%; **Figure 5A**). In the REF and the SAL3 treatments, smaller shifts in the bacterial community composition occurred in the experiment, with the SAL6 and the antibiotic (AB) treatment showing stronger treatment effects, especially at day 6.

The bacterial community composition in the SAL6 treatment was characterized by a high abundance of a flavobacterial OTU (**Figure 4B**). This OTU was also present in high abundances in other treatments and dominated the natural sites but with lower abundances than the experiment samples. In the salinity 6 treatment, hydrogenophagal OTUs became most abundant, whereas in the SAL3 aquaria a smaller increase was observed. The chitinobacterial LD29 was also abundantly found in the treatment, by having the highest abundance in SAL6 on day 6. The genera *Limnohabitans* and *Fluvicola* were more abundant in the REF and SAL3 treatments, whereas in the SAL6 and AB treatments they had a reduced prevalence. In addition, in both salinity treatments *Candidatus* Symbiobacter was among the most abundant bacteria. In the AB manipulation, *Sediminibacterium*, *Taibaiella*, *Paucibacter*, and *Sphingorhabdus* were resistant to the AB treatment, whereas the numbers of other bacteria were reduced compared with the reference and other treatments. The increase in Chao1 richness estimate in the water bacterial community between day 6 and day 8 (**Figure 1**) was connected with the appearance of highly diverse and low-abundance OTUs (**Supplementary Figure 7**), of which approximately 50% differed between each of the treatments (**Supplementary Table 1**).

Visualization by PCoA of the sediment and water bacterial community, including only day 1 and day 8, revealed a difference between day 1 and day 8 bacterial community composition on the first coordinate, and salinity on the second coordinate, especially for the water (**Supplementary Figures 8A, B**). The plotting of the second coordinate against salinity showed a steeper slope (water day 8: *y* = –0.045*x* + 0.0328; sediment day 8: *y* = –0.035*x* + 0.032) for day 8 water bacterial community composition than the sediment bacterial community composition (**Supplementary Figure 9**). The water bacterial community ANCOVA slopes were not equal (*p* < 0.0001, *F* = 33.8) indicating a stronger salinity effect in day 8 water bacterial community composition than in the sediment bacterial community.

The sediment bacterial communities had a statistically different composition in natural sites with different salinity (PERMANOVA, *R* ^2^ = 44%, *p* < 0.0001; **Figure 3B**). The sediment samples from the temporal freshwater pond (FP) had the highest compositional similarity with the SAL3 site. The Jaccard similarity index value of the freshwater pond bacterial community composition, compared with other freshwater sites, was 61%, with SAL3 and SAL6 sites 66% and 58%, respectively. The pond sediment bacterial community was statistically different from salinity 6 sites (PERMANOVA, *R* ^2^ = 32.8%, *p* < 0.001). The bacterial community in the Nõva sampling site (SAL6) was statistically different from other SAL6 sites (PERMANOVA, *R* ^2^ = 67%, *p* < 0.05) indicating compositional variability in the littoral area with the same salinity. The cDNA-based analysis showed overlap in the sediment bacterial communities in FW, FP, and SAL3 sites leading to a statistically significant differentiation between sites having freshwater (FW and FP) and SAL6 sites (PERMANOVA, *R* ^2^ = 66%, *p* < 0.001). In the natural sediment sites and in the experiment, *Flavobacterium*, *Limnohabitan*s, and *Fluvicola* were the most abundant genera in the FW sites, but not in the pond, and their numbers decreased in the higher salinity (**Figure 4A**). *Pseudarcicella* and the HGC-I clade were also abundant in FW and had very low abundances in the FP, SAL3, and SAL6 sites. The freshwater pond was differentiated from the other sites by the higher numbers of *Tabrizicola* and *Arenimonas* and the shared abundant genera, such as *Hydrogenophaga*, *Luteolibacter*, and unclassified Rhodobacteraceae with the saline sites. In addition, salinity 3 had more *Pseudorhodobacter*, *Hydrogenophaga*, and unclassified Saprospiraceae in abundance, whereas in the salinity 6 sites *Ilumatobacter*, unclassified Desulfocapsaceae, and *Rhodopirellula* predominated. When the natural and treatment sites were compared, sediment bacterial communities from similar salinity sites did not group together. The PERMANOVA test validated statistically significant differences for SAL6 natural and experimental bacterial communities (**Figure 3C**; PERMANOVA *R* ^2^ = 34%, *p* < 0.05).

The DNA- and cDNA-based bacterial community composition in the water sampled on the natural sites did not differ significantly (PERMANOVA *p* > 0.05) between the different salinities. The comparison of natural water sites and experiment bacterial communities with the respective salinity resulted with no clustering by salinity groups (**Figure 5C**).

The genera *Flavobacterium*, *Limnohabitans*, and *Fluvicola* were the most abundant OTUs in the natural sites (**Figure 4B**). The most abundant bacteria in the FW and SAL3 sites were very similar (**Figure 5B**). A very different bacterial community was found in the freshwater pond. The dominating genera were *Aphanizomenon* NIES81, *Anabaena* BECID22, *Clostridium* sensu stricto 1, *Polaribacter*, and *Hydrogenophag*a.

## Discussion

Salinity is among the most important factors in structuring bacterial communities (Lozupone and Knight, 2007). Current climate change and anthropogenic activities cause freshwater areas to become saline. This can occur as a pulse disturbance during an extreme weather event (IPCC, 2022) or as a result of anthropogenic activities, including industrial salt discharge (Cañedo-Argüelles et al., 2013). Alternatively, slower salinization occurs by sea level rise (IPCC, 2022), increased evaporation, and lower precipitation (Jeppesen et al., 2020), or via permanent industrial salt discharge (Bäthe and Coring, 2011). The aim of this study was to compare salinization effects on sediment and water bacterial communities in experimental setups and in natural sites to investigate if salinity disturbance alters the bacterial community composition and richness. Salinization occurs by mixing freshwater with saltier water, which results in brackish water typically characterized by a salinity range 3–26. We focused on the salinity range 0.2–6.0, which covers the first stage in freshwater salinization. Comparable to our experimental setup, a previous study used salinities of 3.3, 5.7, and 8.7 to mimic industrial salt discharges (Cañedo-Argüelles et al., 2013). The 2022 ecological disaster, caused by industrial salt discharge in the Oder River was also within the same salinity range (Free et al., 2023).

The first hypothesis, that an increase in salinity alters the bacterial community composition, was only partially proven in this study. An increase in salinity did not cause significant changes in the sediment bacterial community composition in the experiment (**Figure 3A**). However, for the water bacteria, a significant effect was observed (**Figure 5A**). This suggests that sediment bacterial communities are more resistant to these moderate pulse salinity disturbances than water bacterial communities. However, a potential effect on the sediment bacterial community due to increased salinity was observed for day 8 in the salinity 6 treatment. Here a change appeared in half of the replicated aquaria, suggesting that salinity 6 could be a tipping point where the sediment bacterial community becomes sensitive. This is supported by a drop in the Chao1 richness estimate after 8 days at salinity 6 (**Figure 1A**). In the well-established sediment bacterial communities in the Baltic Sea, the composition differed between salinity 4 and 8 (Klier et al., 2018). Accordingly, pulse salinity changes exceeding a salinity of 6 could impact sediment bacterial communities. More studies with a higher resolution in this salinity range are needed to evaluate a potential tipping point for sediment bacterial communities. As expected, the addition of antibiotics 1,000× stronger than the typical concentrations found in the environment (usually in the low ng/L range; Larsson, 2014) caused a significant disturbance in the sediment bacterial community composition and richness. Therefore, intense disturbance also results in changes in the sediment bacterial community. In contrast to the resistance against a pulse increase in salinity in the experiment, the littoral sediment bacterial community composition in rivers and coastal salinity 3 and 6 sites clustered according to salinity (**Figure 3B**). This is similar to previous studies that have found a significant separation of offshore sediment bacterial communities according to salinity (Yang et al., 2016; Klier et al., 2018). Hence, sediment bacterial communities are adapted to specific salinities during permanent exposure.

A significant salinization effect and a strong disturbance of antibiotics to the water bacterial community was observed in the experiment (**Figure 5A**) showing a sensitivity to different types of pulse disturbances. The responses of the water bacterial communities to the alteration of salinity (Langenheder et al., 2003) and antibiotics (Eckert et al., 2019) have been shown previously. The missing clustering of water bacterial community composition in natural sites according to salinity (**Figure 5B**) suggests that the littoral bacterial communities were strongly influenced by local factors, including macrophytes (Duarte et al., 2005), nutrient concentration, and terrestrial effects (Figueroa et al., 2021). This supports the hypothesis that water bacterial communities were sensitive to complex mixtures of environmental factors that were stronger than the effect of salinity alone. However, as we focused on the effect of salinity, we can only speculate about the impact of the other parameters. In addition, historical site-specific events and temporal changes in salinity could have altered the development of the bacterial community in the water (Renes et al., 2020; Philippot et al., 2021). In contrast to the complex mixture of changing factors in the environment, the variability of these factors was minimized and similar in all aquaria in the experiment thus allowing us to focus on the effect of salinity to bacterial communities alone.

The second hypothesis about the reduction of bacterial richness during salinity disturbance can only partially be proven as the water bacterial richness changed significantly in the experiment but sediment bacterial richness did not. A decrease in the bacterial richness is a typical response of aquatic bacterial communities to pulse disturbance (Allison, 2004; Downing and Leibold, 2010; Renes et al., 2020). However, a decrease in the water bacterial richness was observed in parallel in all aquaria, including in the reference. This was a reaction to the confined artificial aquaria conditions where there was an altered nutrient availability (Ionescu et al., 2015; Herlemann et al., 2017; Herlemann et al., 2019) and different grazing patterns of bacteria by ciliates and heterotrophic nanoflagellates (Ionescu et al., 2015; Herlemann et al., 2019). A significantly stronger dynamic of bacterial richness was observed for the more intense manipulation (i.e., SAL6 and AB) supporting a sensitivity of the water bacterial richness to pulse disturbance in addition to confinement stress. Disturbance intensity is a key determining how water bacterial communities respond to disturbance (Berga et al., 2012; Philippot et al., 2021; Kivistik et al., 2022). In salinization experiments of rock pools (salinity range 3–12), bacterial communities changed at an increasing magnitude with increasing salinities (Berga et al., 2017). In contrast to pulse stress, bacterial richness has been shown to be constant at different salinity levels in permanent brackish water environments such as in the Baltic Sea (Herlemann et al., 2011; Herlemann et al., 2016). Similar results were found in our study for natural sites with different salinity (**Figure 2**). Hence, our results suggest that water bacterial communities are reacting to strong pulse disturbances with decreased species diversity and changes in the bacterial community composition. A longer exposure to different salinity caused the adaptation of the bacterial community resulting in comparable richness. However, a reduced water bacterial richness in permanent exposure to the salinity can be found in hypersaline lakes (Ji et al., 2019) suggesting that this is only applicable to the brackish environment.

The OTUs abundantly found in the sediment and water, including *Flavobacterium*, *Hydrogenophaga*, and *Limnohabitans*, were also abundant in the experiment. They are typical aquatic bacteria, of which the *Flavobacterium* species are known members of freshwater but also the Baltic Sea (Klier et al., 2018). Water bacteria of the genus *Limnohabitans* are capable of utilizing algal exudates and can survive well in artificial environments (Pérez and Sommaruga, 2006; Jezberová et al., 2017). The abundance of *Hydrogenophaga*, a genus containing many hydrogen-oxidizing bacteria, increased with increased salinity. In contrast, the abundance of the HGC-I OTU was high in natural water sites, except the pond, but was significantly lower in the experiment, as this bacterial group is known to be sensitive to artificial environments (Herlemann et al., 2019).

The increase in salinity did not change the sediment bacterial community in the experiment severely. The bacteria abundant during the experiment (unclassified Comamonadaceae, unclassified Steroidobacteriaceae, *Chthoniobacter*, Bacteroidetes vadinHA17, and *Thiobacillus*) were also among the most abundant sediment bacteria in the natural samples (**Figure 4A**). Aerobic Comamonadaceae have been found in lower salinity concentrations in previous studies (Aguirre et al., 2017). The *Chthoniobacter* genus contains only free-living, aerobic chemoheterotrophic, and metabolizing organic carbon species found in soil and freshwater sediments (Sangwan et al., 2004; MGnify, 2019). The majority of the available vadinHA17 sequences in the SILVA NR database have been obtained from sediments and anaerobic bioreactors where proteinaceous substrates are abundant and these bacteria are most likely to be capable of protein and amino acids degradation (Mei et al., 2020). The data of salinity tolerance for the vadinHA17 group suggest an adaptation to brackish environments (Klier et al., 2018). This is in accordance with our results where the abundance did not decrease up to a salinity of 6. *Thiobacillus* species have been found to dominate surface sediments in freshwater lake–river systems (Duan et al., 2020). In a salinity of 6, the *Chloroflexi* group KD4–96 also became abundant. This group has been found in deep-sea sediments, but also in the Bothnian Sea with a similar salinity to that which was used in our experiment (Rasigraf et al., 2020). Several of the most abundant OTUs that tolerated the increase in salinity were also found in the antibiotic treatment (e.g., *Hydrogenophaga*, *Limnohabitans*, *Arenimonas*, *Luteoliobacter*) but in lower abundances. The differences in salinity of natural site sediments were enough to facilitate different dominating bacteria. In a salinity of 3, the genera *Hydrogenophaga*, *Flavobacterium*, and *Pseudorhodobacter*, and unclassified Saprospiraceae predominated. The salinity 6 samples from the envrionment and the experiment contained bacteria found in brackish water sediments with both aerobic and anaerobic metabolisms, such as *Ilumatobacter* (aerobic heterotroph) (Fang et al., 2015), unclassified Desulfocapsaceae (anaerobic respiration) (Galushko and Kuever, 2021), and *Rhodopirellula* (chemoheterotrophic aerobes) (Sreya et al., 2023).

In addition to *Limnohabitans*, *Flavobacterium*, and HGC-I, the verrucomicrobial lineage LD29 were highly abundant in the experiment water but had very low abundance in the natural sites. Previously these bacteria have been found in a similar salinity range in the Baltic Sea (Herlemann et al., 2013) but have strong seasonal dynamics (Lindh et al., 2015) and were therefore possibly missed in our natural samples. In the water bacterial community, antibiotic treatment supported the dominance of *Sphingorhabdus*, *Sediminibacterium*, *Taibaiella*, and *Paucibacter*, which are probably more resistant to the added antibiotics. Hence, the Chao1 richness recovery in the antibiotic treatment is probably connected with the ability of the bacteria to proliferate in the presence of the antibiotics used.

Despite the overlaps of the OTUs between the experimental and natural sites, there was a significant difference in the overall bacterial community composition (**Figures 3C**, **5C**). An important difference between the natural site and experiment are the abundances of the bacteria but also the less abundant OTUs.

An exceptional salinity-driven bacterial community was found in the natural pond situated on the shoreline of the Baltic Sea. It is located in close proximity to the outflow from a freshwater river. The pond contained freshwater during sampling, whereas the investigated sediment bacterial community had a high similarity to the brackish coastal bacterial communities (**Figure 3B**). The pond water bacterial community composition clustered randomly. We hypothesize that the pond was filled by freshwater due to the natural shift of the river outflow in the spring (i.e., 4 months previous to the sampling), but the sediment was originally part of the brackish Baltic Sea shoreline. Therefore, the brackish sediment bacterial community was overlaid with freshwater but contained its natural brackish sediment bacteria. As a result, the pond had a brackish sediment bacterial community despite being filled with freshwater. This supports the resistance of sediment bacterial communities to changes in salinity possibly for several months in the environment. The freshwater pond sediment was different from the other sites by the increased abundance of *Tabrizicola*, *Arenimonas*, and the facultative anaerobe *Rhodoferax*. The genus *Tabrizicola* consists mostly of chemotrophic bacteria but also has members with anoxygenic photosynthetic capabilities that have been isolated from freshwater or saline lake sediment (Liu et al., 2019; Tarhriz et al., 2019). The genus *Arenimonas*, which is abundant in the sediment of the freshwater pond and salinity 3 site, has been previously isolated from soil (Han et al., 2020) but also from seashore sand (Kwon et al., 2007). Most known members of the genus *Rhodoferax* are anoxygenic photoheterotrophs or organomixotrophs with capability to reduce Fe(III) (Finneran et al., 2003). The pond sediment shared abundant genera, such as *Hydrogenophaga* and *Luteolibacter*, and unclassified Rhodobacteraceae with saline site sediments, reflecting its history of being in contact with the Baltic Sea. Hence a brackish photoheterotrophic bacterial community seems to be characteristic for this pond.

During the last decades, a number of studies have investigated the effects of disturbance on the bacterial community composition with the conclusion that bacterial communities are in most cases sensitive to disturbances (Allison and Martiny, 2008; Shade et al., 2012; Griffiths and Philippot, 2013; Lindh and Pinhassi, 2018). Disturbance may change species richness and bacterial community composition, which may in turn have consequences for the stability of ecosystem processes (Langenheder et al., 2006; Peter et al., 2011; Reed and Martiny, 2013; Gibbons et al., 2016; Berga et al., 2017; Renes et al., 2020). The effect of disturbance depends on its intensity, disturbance type, frequency, and length, as well as the species tolerance capacity (Shade et al., 2012; Gibbons et al., 2016; Eckert et al., 2019). Compositional resistance of water bacterial communities has been shown to decrease with increasing salinity levels (Berga et al., 2017). Contrary to this, our study showed that the sediment bacterial community was resistant and buffered pulse disturbance in the salinity range tested. A permanent exposure to natural brackish conditions caused a selection of sediment bacteria without loss in species richness. This represents a long-term adaptation of the brackish sediment bacterial communities that perform the ecosystem processes. A resistance of sediment bacteria to changes in salinity has been shown previously (Reed and Martiny, 2013). The reason for the resistance of bacterial communities to salinification has been connected with fluctuating environments (Berga et al., 2017). However, since the origin of the water and sediment used in our experiment was a freshwater lake that has not been in contact with saltwater since the last ice age, adaptation to salt fluctuations cannot explain the observed resistance. Sediment bacteria are dispersal limited due to the physical barrier of the sediments. This dispersal limitation could be a reason for the higher resistance of sediment bacterial communities toward salinity disturbance. The importance of dispersal to bacterial communities in aquatic environments has been shown in several studies (Jones and McMahon, 2009; Lindström and Östman, 2011; Martiny et al., 2011; Székely et al., 2013). In the environment, dispersal can supply bacteria that are better adapted to a disturbed environment. The example of the pond shows that several months of being exposed to freshwater was insufficient for a shift in the brackish sediment bacterial community composition. The laboratory experiments, in contrast, excluded effects of natural dispersal that were present in the environment (Lennon and Jones, 2011). Together with the limited time, dispersal limitation of the sediment resulted in the resistance of the sediment bacterial community to salinity pulse changes. Moreover, a similar bacterial richness in the sediment based on DNA and cDNA suggests a limited seedbank of bacteria available that could respond to changes in salinity. These dormant bacteria are a valuable seedbank, which consists of individuals capable of being resuscitated after or during disturbance (Lennon and Jones, 2011). The pulse salinity disturbances had a significant effect on the water bacterial richness and community composition in the experiments. Also, for the water bacterial community, the laboratory experiment excludes the effects of natural dispersal. The cDNA-to-DNA ratio was much lower in the water than in the sediment, suggesting that a large water bacterial seedbank provides a source for shifts in the bacterial community. Finally, neither the sediment nor the water bacterial community in the experiment resembled the natural brackish bacterial communities after changes in salinity (**Figures 3C**, **5C**). This supports the important role of a seedbank during disturbance and also suggests a biogeography for bacterial communities.

We conclude that sediment bacterial communities are more resistant to pulse salinization than the water bacterial communities. The resistance of sediment bacteria may be caused by dispersal limitations due to the physical structure of the sediment and a smaller seedbank in our experiment. However, a longer exposure to salinity and dispersal, as experienced in the environment, results in an adaptation of the sediment bacterial communities. Moreover, stronger effects such as antibiotics indicate that other stressors than salinity may evade the resistance of sediment bacterial communities during pulse disturbance. For the management of industrial salt disposal and climate change, this implies a resistance of sediment bacterial communities to mild short-term salinization events, possibly without significant effects on ecosystem functions. For water bacterial communities significant responses to changes in salinity are expected.

## Data availability statement

The datasets presented in this study can be found in online repositories. The names of the repository/repositories and accession number(s) are as follows: https://www.ncbi.nlm.nih.gov/, bioproject PRJNA724976 accession numbers SAMN18865857-SAMN18866052.

## Author contributions

All authors performed the experiments and sampling. HT and CK did DNA extraction and preparation for sequencing. HT, VK, and DH did statistical and bioinformatics analysis. HT submitted sequences. HT wrote the original draft. HT, DH, CK, and VK contributed to writing, reviewing, and editing of the manuscript. All authors contributed to the article and approved the submitted version.
