# Supplementary material for: Resistance of freshwater sediment bacterial communities to salinity disturbance and the implication for industrial salt discharge and climate change-based salinization
Source: Front Microbiomes. 2023 Nov 13;2:1232571. doi: 10.3389/frmbi.2023.1232571 (PMC12993645; doi:10.3389/frmbi.2023.1232571)
Supplement: Supplementary file 1 [file DataSheet_1.pdf]

Resistance of freshwater sediment bacterial communities to salinity disturbance and implication for industrial salt discharge and climate change based salinization

Helen Tammert[1,2], Carmen Kivistik[1], Veljo Kisand[1,2], Kairi Käiro[1], Daniel P. R. Herlemann[1,3]*

[1]Estonian University of Life Sciences, Chair of Hydrobiology and Fishery, Centre for Limnology, EE61101 Elva municipality, Tartu County, Estonia

[2]Institute of Technology, University of Tartu, 50411 Tartu, Estonia

[3]Leibniz Institute for Baltic Sea Research Warnemünde, Seestraße 15, 18119 Rostock

* Correspondence to daniel.herlemann@io-warnemuende.de

Supplementary Table 1. Most abundant OTUs that appear in treatments on day 8 compared to day 6 of the experiment. REF: reference, FW: freshwater, SAL3: salinity 3, SAL6: salinity 6, AB: antibiotic treatment. The reads were averaged between the replicate samples.

OTU	Reads	Treatment
Bacteria;Proteobacteria;Gammaproteobacteria;Burkholderiales;Methylophilaceae;uncultured	22.0	REF
Bacteria;Proteobacteria;Alphaproteobacteria;Rhodobacterales;Rhodobacteraceae;Roseivivax	12.5	REF
Bacteria;Myxococcota;Polyangia;Polyangiales;BIrii41	10.5	REF
Bacteria;Proteobacteria;Gammaproteobacteria;Burkholderiales;Comamonadaceae;Methylibium	10.0	REF
Bacteria;Bacteroidota;Bacteroidia;Flavobacteriales;Crocinitomicaceae;uncultured	9.5	REF
Bacteria;Proteobacteria;Gammaproteobacteria;Burkholderiales;Nitrosomonadaceae;Nitrosomonas	8.0	REF
Bacteria;Proteobacteria;Alphaproteobacteria;Sphingomonadales;Sphingomonadaceae;Altererythrobacter	7.5	REF
Bacteria;Actinobacteriota;Actinobacteria;Nitriliruptorales;Nitriliruptoraceae;Egicoccus	6.0	REF
Bacteria;Proteobacteria;Alphaproteobacteria;Caulobacterales;Hyphomonadaceae;Oceanicaulis	6.0	REF
Bacteria;Proteobacteria;Gammaproteobacteria;HOC36	6.0	REF
Bacteria;Actinobacteriota;Acidimicrobiia;Microtrichales;Microtrichaceae;IMCC26207	6.7	SAL3
Bacteria;Actinobacteriota;Actinobacteria;Micrococcales;Microbacteriaceae;uncultured	6.7	SAL3
Bacteria;Bacteroidota;Bacteroidia;Sphingobacteriales;Sphingobacteriaceae;Sphingobacterium	6.0	SAL3
Bacteria;Proteobacteria;Gammaproteobacteria;Pseudomonadales;Moraxellaceae;uncultured	5.3	SAL3
Bacteria;Acidobacteriota;Acidobacteriae;Bryobacterales;Bryobacteraceae;Bryobacter	5.0	SAL3
Bacteria;Proteobacteria;Gammaproteobacteria;Piscirickettsiales;Piscirickettsiaceae;Candidatus Endoecteinascidia	4.3	SAL3
Bacteria;Fusobacteriota;Fusobacteriia;Fusobacteriales;Fusobacteriaceae;Fusobacterium	3.3	SAL3
Bacteria;Desulfobacterota;Desulfovibrionia;Desulfovibrionales;Desulfomicrobiaceae;Desulfomicrobium	3.0	SAL3
Bacteria;Proteobacteria;Alphaproteobacteria;Rhodospirillales;Rhodospirillaceae;Novispirillum	3.0	SAL3
Bacteria;Bacteroidota;Bacteroidia;Bacteroidales;Marinilabiliaceae;uncultured	2.7	SAL3
Bacteria;Patescibacteria;Gracilibacteria;JGI 0000069-P22	16.3	SAL6
Bacteria;Proteobacteria;Gammaproteobacteria;Xanthomonadales;Xanthomonadaceae;Stenotrophomonas	8.8	SAL6
Bacteria;Actinobacteriota;Actinobacteria;Micrococcales;Microbacteriaceae;Chryseoglobus	8.0	SAL6
Bacteria;Proteobacteria;Alphaproteobacteria;Rhizobiales;Rhizobiales Incertae Sedis;Anderseniella	8.0	SAL6
Bacteria;Proteobacteria;Gammaproteobacteria;Xanthomonadales;Rhodanobacteraceae;Dokdonella	7.5	SAL6
Bacteria;Proteobacteria;Alphaproteobacteria;uncultured	7.3	SAL6
Bacteria;Actinobacteriota;Actinobacteria;Propionibacteriales;Nocardioidaceae;Aeromicrobium	6.0	SAL6
Bacteria;Proteobacteria;Alphaproteobacteria;Caulobacterales;Caulobacteraceae;uncultured	5.8	SAL6
Bacteria;Firmicutes;Clostridia;Peptostreptococcales-Tissierellales;JTB215	5.3	SAL6
Bacteria;Proteobacteria;Gammaproteobacteria;Burkholderiales;Aquaspirillaceae;Laribacter	5.0	SAL6
Bacteria;Bacteroidota;Bacteroidia;Bacteroidales;Bacteroidaceae;Bacteroides	120.0	AB
Bacteria;Proteobacteria;Gammaproteobacteria;Burkholderiales;Rhodocyclaceae;Azoarcus	33.0	AB
Bacteria;Proteobacteria;Gammaproteobacteria;Burkholderiales;Comamonadaceae;Variovorax	25.0	AB
Bacteria;Proteobacteria;Gammaproteobacteria;Burkholderiales;Oxalobacteraceae;Massilia	17.0	AB
Bacteria;Proteobacteria;Gammaproteobacteria;Burkholderiales;Comamonadaceae;Methylibium	16.0	AB
Bacteria;Gemmatimonadota;Gemmatimonadetes;Gemmatimonadales;Gemmatimonadaceae;uncultured	13.0	AB
Bacteria;Proteobacteria;Alphaproteobacteria;Rhizobiales;Xanthobacteraceae;Bradyrhizobium	12.0	AB

Bacteria;Planctomycetota;Planctomycetes;Gemmatales;Gemmataceae;Gemmata	11.0	AB
Bacteria;Proteobacteria;Gammaproteobacteria;Burkholderiales;Methylophilaceae;Methylotenera	10.0	AB
Bacteria;Proteobacteria;Gammaproteobacteria;Enterobacterales;Pseudoalteromonadaceae;Pseudoaltero monas	10.0	AB

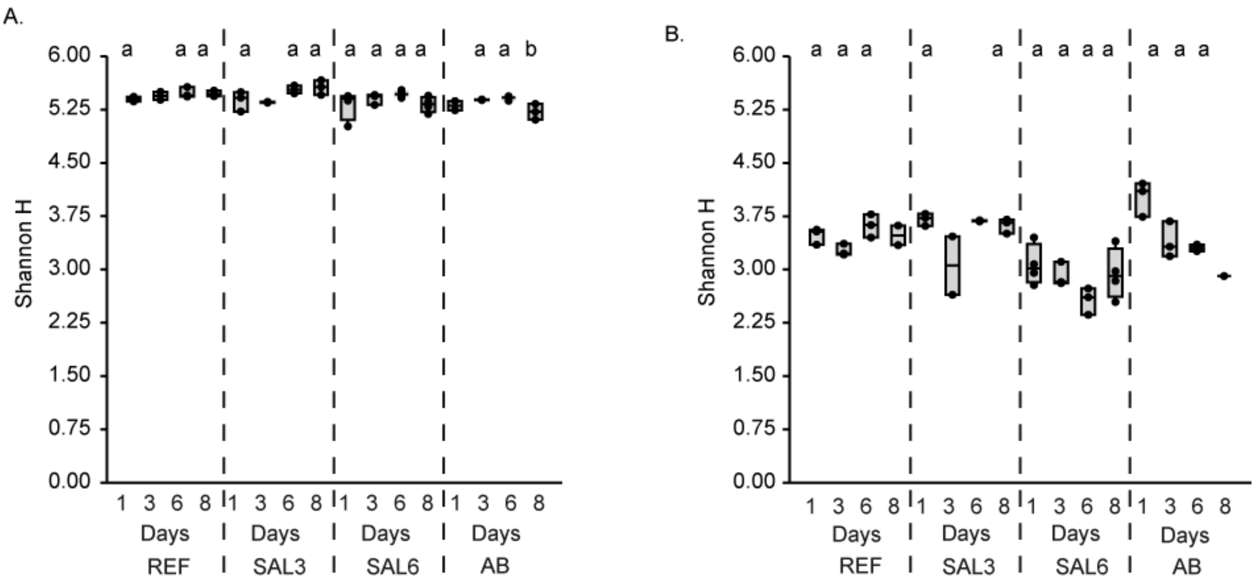

Supplementary Figure 1. The bacterial richness based on DNA represented by Shannon index in the reference and treatment during the time course of the experiment (A) sediment, (B) water. REF: reference aquaria, SAL3: salinity increased to 3, SAL6: salinity increased to 6, AB: antibiotic treatment. Non-capital letters (a, b) above the box-plots indicate statistical significance ($p<0.05$) within one group that were separated by dashed lines.

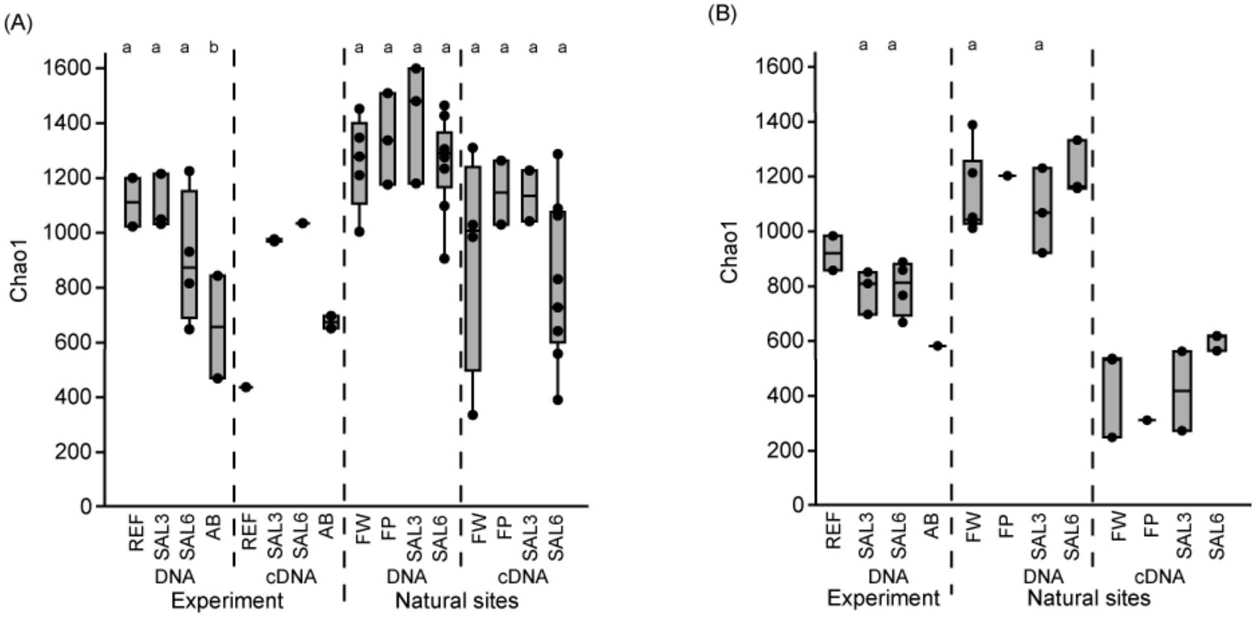

Supplementary Figure 2. The bacterial Chao1 richness based on the DNA and cDNA on the last day (day 8) of the experiment and in the natural sites. **(A)** sediment, **(B)** water. Experiment: REF: reference aquaria, SAL3: salinity increased to 3, SAL6: salinity increased to 6, AB: antibiotic treatment. Natural sites: FW: freshwater, FP: freshwater pond, SAL3: salinity 3, SAL6 salinity 6. Non-capital letters (a, b) above the box-plots indicate statistical significance within one group that are separated by dashed lines.

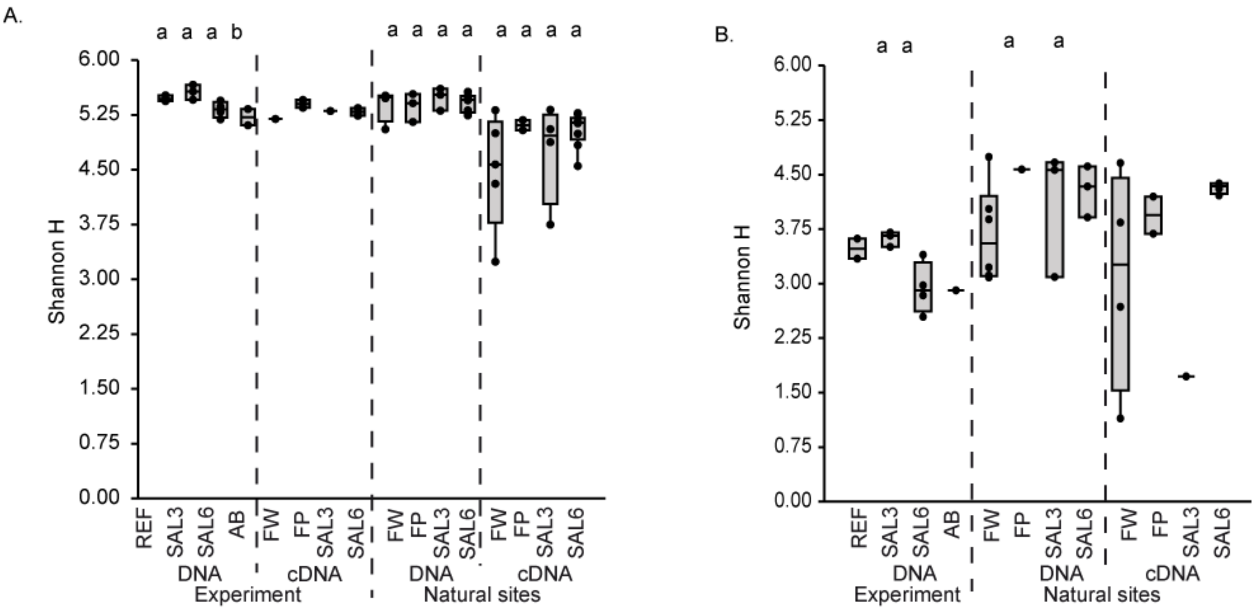

Supplementary Figure 3. The bacterial richness represented by Shannon index based on the DNA and cDNA on the last day (day 8) of the experiment and in the natural sites. **(A)** sediment, **(B)** water. Experiment: REF: reference aquaria, SAL3: salinity increased to 3, SAL6: salinity increased to 6, AB: antibiotic treatment. Natural sites: FW: freshwater, FP: freshwater pond, SAL3: salinity 3, SAL6 salinity 6. Non-capital letters (a, b) above the box-plots indicate statistical significance within one group that are separated by dashed lines.

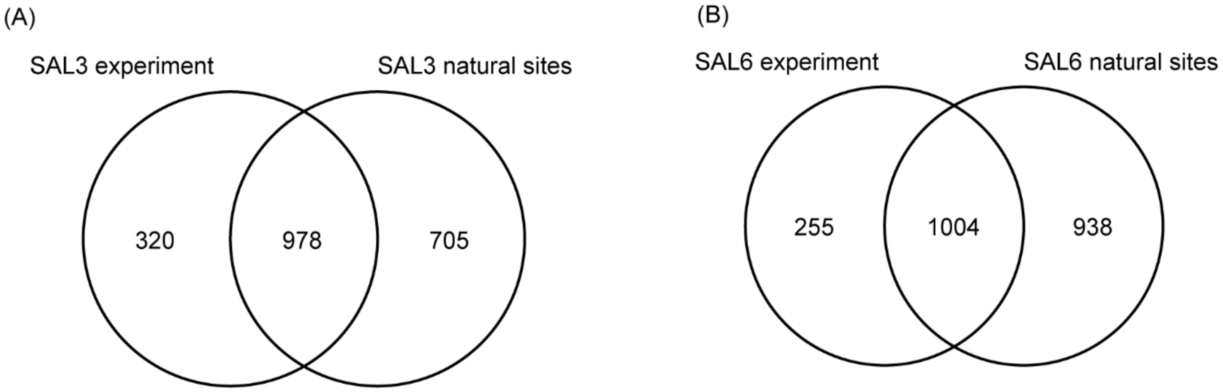

Supplementary Figure 4. Shared and specific OTUs of the sediment bacterial community in the experiment on day 8 and natural sites based on DNA. (A) salinity 3, (B) salinity 6.

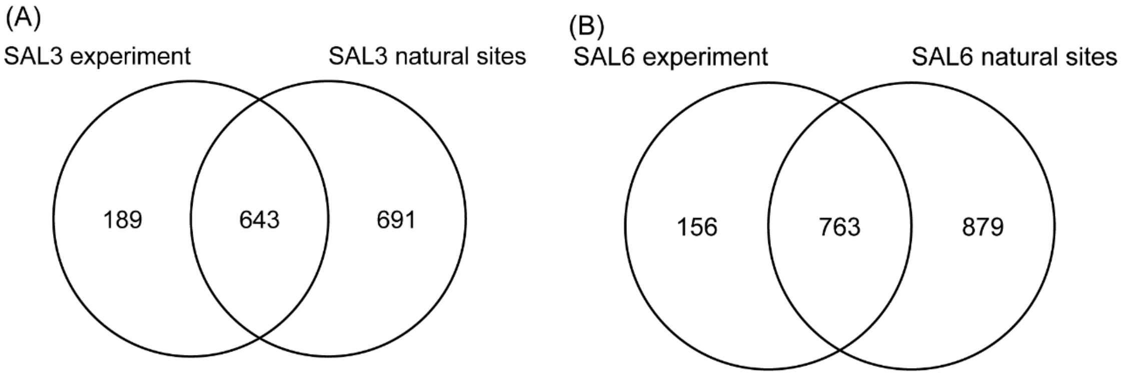

Supplementary Figure 5. Shared and specific OTUs of the water bacterial community in the experiment day 8 and natural sites based on DNA. (A) salinity 3, (B) salinity 6.

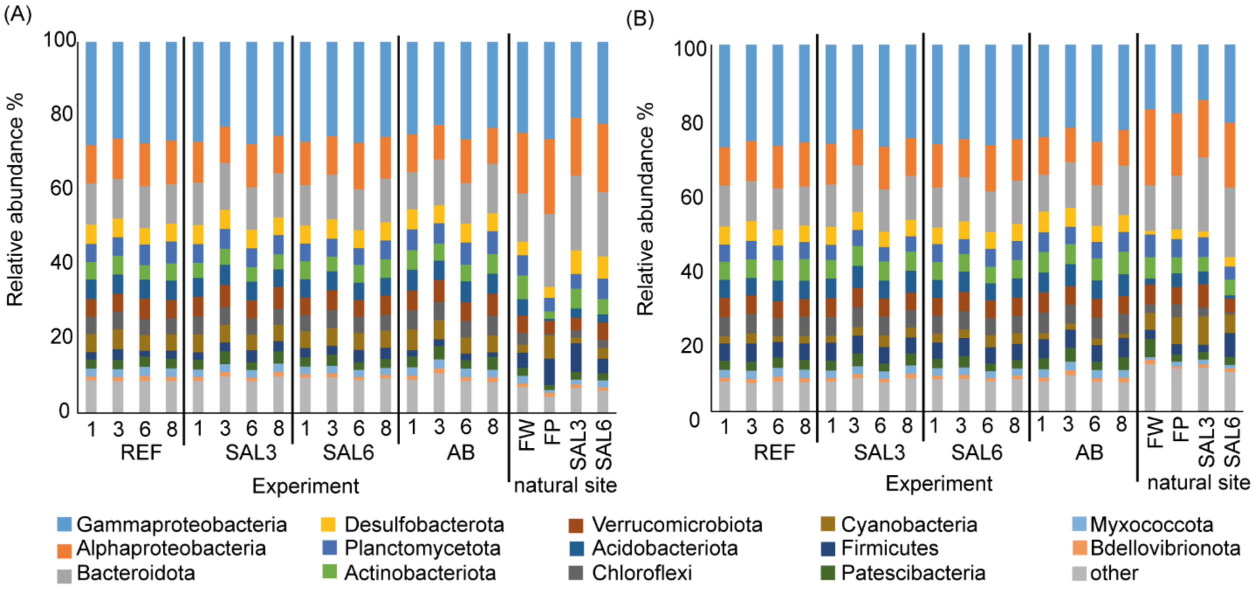

Supplementary Figure 6. Bacterial community composition on Phylum/Class level during the experiment and in the natural sites. (A) sediment (B) water. Experiment: REF: reference aquaria, SAL3: salinity increased to 3, SAL6: salinity increased to 6, AB: antibiotic treatment. Natural sites: FW: freshwater, FP: freshwater pond, SAL3: salinity 3, SAL6 salinity 6. The number indicates the day of the experiment.

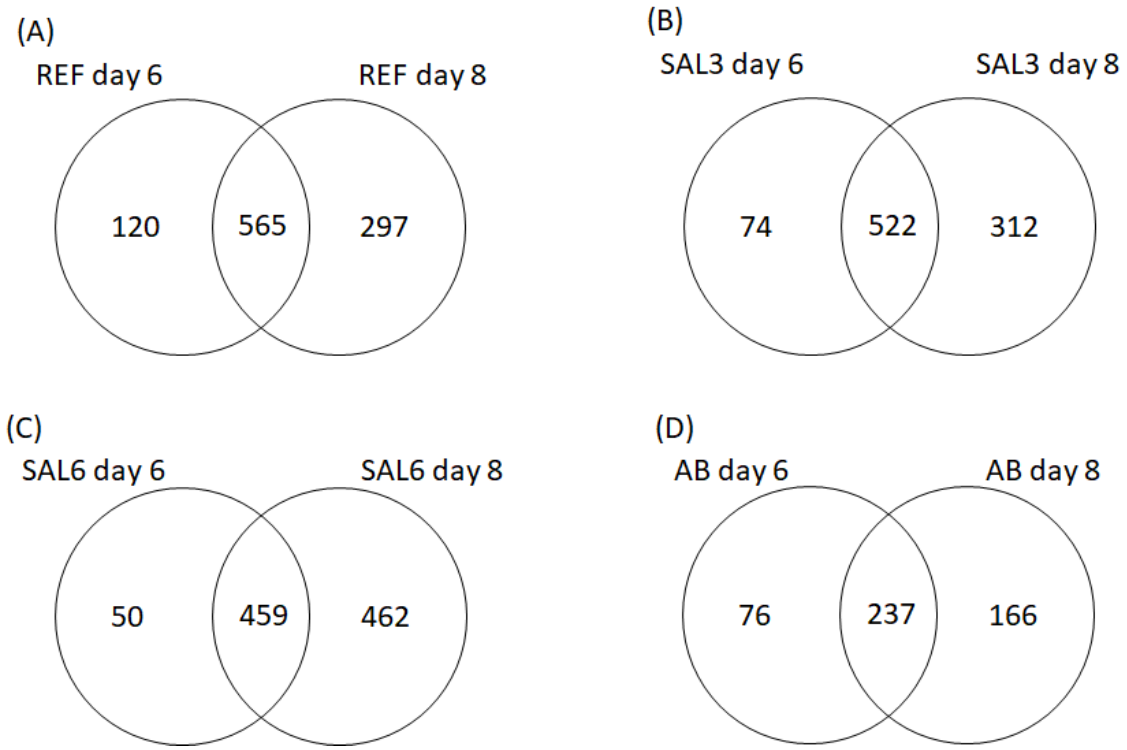

Supplementary Figure 7. Shared and specific OTUs of the water bacterial community in the experiment day 6 and day 8 based on DNA. (A) REF: reference, (B) salinity 3: SAL3, (C) salinity 6: SAL6 and (D) antibiotic: AB.

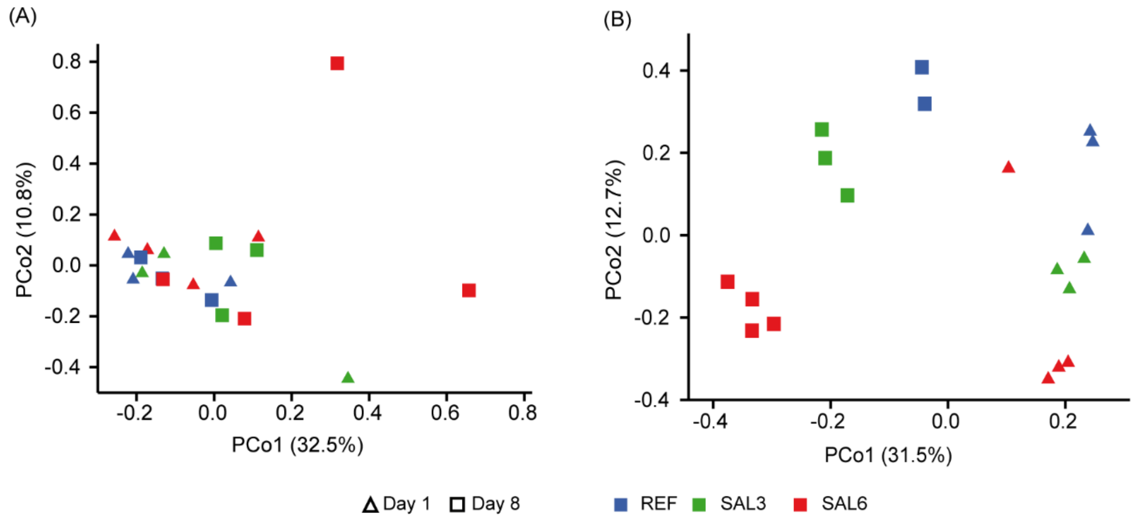

Supplementary Figure 8. Bacterial community composition on day 1 and day 8 of the experiment. (A) sediment, (B) water. REF: reference aquaria, SAL3: salinity increased to 3, SAL6: salinity increased to 6.

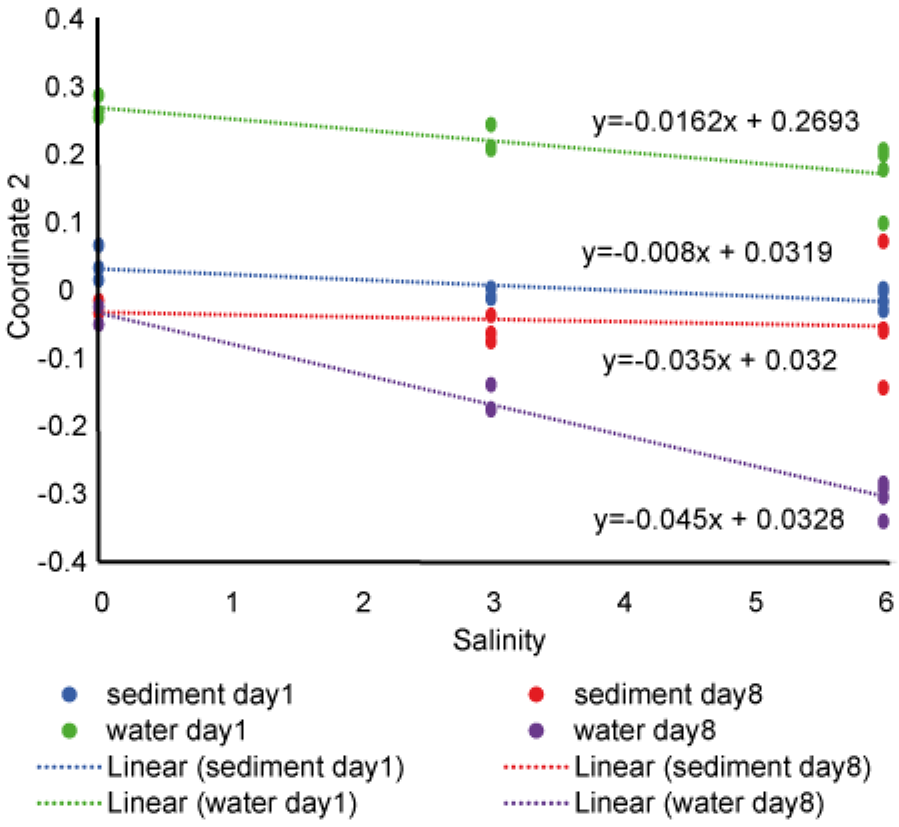

Supplementary Figure 9. Plot of the second coordinate of sediment and water bacterial community composition second coordinate in PCo analysis on day 1 and day 8 against salinity based on Supplementary Figure 8.
